# Single vs. dual antiplatelet therapy in patients with severe peripheral arterial disease undergoing transcatheter aortic valve implantation: insights from the Hostile registry

**DOI:** 10.1093/ehjcvp/pvaf049

**Published:** 2025-07-15

**Authors:** Mattia Galli, Roberto Nerla, Fausto Castriota, Francesco Saia, Won-Keun Kim, Alessandro Iadanza, Ole De Backer, Francesco Burzotta, Nicolas M Van Mieghem, Thomas Pilgrim, Giuseppe Musumeci, Max M Meertens, Michael Joner, Francesco Meucci, Stefan Toggweiler, Luca Testa, Sergio Berti, Matteo Montorfano, Daniel Braun, Marco De Carlo, Marco Barbanti, Giulio Stefanini, Georg Nickenig, Tommaso Piva, Azeem Latib, Italo Porto, Ran Kornowski, Antonio L Bartorelli, Mohamed Abdel-Wahab, Tullio Palmerini

**Affiliations:** Department of Medical-Surgical Sciences and Biotechnologies, Sapienza University of Rome, Latina 04100, Italy; Maria Cecilia Hospital, GVM Care and Research, Cotignola 48033, Italy; Maria Cecilia Hospital, GVM Care and Research, Cotignola 48033, Italy; Maria Cecilia Hospital, GVM Care and Research, Cotignola 48033, Italy; Cardiology Unit, Cardiac Thoracic and Vascular Department, IRCCS Azienda Ospedaliero-Universitaria di Bologna; Cardiac Thoracic and Vascular Department, Università di Bologna; Leiter interventionelle Herzklappentherapie Kerckhoff-Klinik GmbH Herz und Thorax Zentrum, Bad Nauheim, Germany; UOSA Cardiologia Interventistica Azienda Ospedaliera Universitaria Senese, Siena, Italy; The Heart Center, Rigshospitalet, Copenhagen University Hospital, Copenhagen, Denmark; U.O.C. di Interventistica Cardiologica e Diagnostica Invasiva, Fondazione Policlinico Universitario Agostino Gemelli IRCCS, Rome, Italy; Università Cattolica del Sacro Cuore, Rome, Italy; Department of Cardiology, Thoraxcenter, Erasmus University Medical Center, Netherlands; Inselspital, Bern University Hospital, University of Bern, Switzerland; A.O. Mauriziano Umberto I Hospital, Turin, Italy; University Hospital Cologne—Heart Center, Klinik III für Innere Medizin—Kardiologie, Pneumologie und Internistische Intensivmedizin, Cologne, Germany; German Heart Centre Munich/Deutsches Herzzentrzum München—Munich, Germany; Department of Clinical and Experimental Medicine, Structural Interventional Cardiology, University Hospital Careggi, Florence, Italy; Heart Center Lucerne, Cardiology, Luzerner, Switzerland; Coronary Revascularisation Unit, IRCCS Policlinico S. Donato, San Donato Milanese, Italy; Unit of Diagnostic and Interventional Cardiology, C.N.R. Reg. Toscana G. Monasterio Foundation, Ospedale del Cuore, Massa, Italy; School of Medicine, Vita-Salute San Raffaele University, Milan, Italy; Interventional Cardiology Unit—IRCCS San Raffaele Scientific Institute, Milan, Italy; Department of Medicine I, University Hospital Munich, Medical Faculty—LMU, Munich, Germany; Cardiothoracic and Vascular Department, Pisa University Hospital, Pisa, Italy; Università Degli Studi di Enna ‘Kore,’ Enna, Italy; Division of Cardiology, Ospedale Umberto I, ASP 4 di Enna, Enna, Italy; Department of Biomedical Sciences, Humanitas University, Pieve Emanuele - Milan, Italy; IRCSS Humanitas Research Hospital, Rozzano - Milan, Italy; Medizinische Klinik und Poliklinik II, Herzzentrum Bonn—Universitätsklinikum Bonn, Bonn, Germany; Azienda Ospedaliero-Universitaria, Ospedali Riuniti Umberto I—GM Lancisi, Torette, Ancona, Italy; Montefiore-Einstein Center for Heart and Vascular Care, Montefiore Medical Center Albert Einstein College of Medicine, Bronx, NY, USA; Cardiovascular Disease Unit, IRCCS Ospedale Policlinico San Martino, Genova, Italy; Department of Internal Medicine, University of Genoa, Genova; Rabin Medical Center, Petah Tikva, Israel; IRCCS Ospedale Galeazzi-Sant’Ambrogio, University of Milan, Milan, Italy; Heart Center Leipzig at University of Leipzig, Leipzig, Germany; Cardiology Unit, Cardiac Thoracic and Vascular Department, IRCCS Azienda Ospedaliero-Universitaria di Bologna; Cardiac Thoracic and Vascular Department, Università di Bologna

**Keywords:** Transcatheter aortic valve implantation, Peripheral artery disease, Single antiplatelet therapy, Dual antiplatelet therapy

## Abstract

**Aims:**

Single antiplatelet therapy (SAPT) has been shown to be a safer alternative to dual antiplatelet therapy (DAPT) in patients without atrial fibrillation (AF) undergoing transcatheter aortic valve implantation (TAVI). However, antithrombotic therapy for TAVI patients with severe peripheral artery disease (PAD) remains an underexplored area. This study aimed to evaluate and compare the outcomes of SAPT and DAPT in this high-risk patient population.

**Methods and results:**

The HOSTILE registry was a multicentre, international, observational study including 1707 consecutive patients with hostile femoral access undergoing TAVI in 28 international centres. Among 573 patients without AF treated through transfemoral or non-thoracic alternative approach, 144 received SAPT and 429 DAPT after TAVI. The primary efficacy endpoint was the propensity-adjusted rate of major adverse cardiovascular events (MACE), a composite of cardiovascular death, myocardial infarction, stroke, or transient ischaemic attack. The primary safety endpoint was the propensity-adjusted rate of major bleeding. Outcomes were reported at 30 days and 12 months. Dual antiplatelet therapy was associated with a non-significant reduction in MACE at 30 days [hazard ratio (HR) 0.74, 95% confidence interval (CI) 0.25–2.18; *P* = 0.59] and at 12 months (HR 0.89, 95% CI 0.35–2.24; *P* = 0.80) compared with SAPT, but with a significant interaction between antiplatelet strategy and PAD severity (*P* = 0.01), suggesting a greater benefit of DAPT in patients with a high PAD severity. Dual antiplatelet therapy was associated with reduced all-cause death at 12 months (HR 0.22, 95% CI 0.10–0.47; *P* < 0.001) but not at 30 days (HR 0.26, 95% CI 0.05–1.22; *P* = 0.09) compared with SAPT. There was no difference in major bleeding at 30 days (*P* = 0.13) or 12 months (*P* = 0.10) between groups. There were no differences between groups in any bleeding at 30 days (*P* = 0.16) or 12 months (*P* = 0.17).

**Conclusion:**

In TAVI patients with severe PAD, DAPT was associated with a trend towards improved outcomes compared with SAPT, particularly in those with higher PAD severity. These findings, including the observed reduction in 1-year mortality with DAPT, warrant further investigation in prospective studies.

## Introduction

Transcatheter aortic valve implantation (TAVI), traditionally performed via the transfemoral artery route, has revolutionized the treatment of severe aortic stenosis offering a less invasive alternative to surgical aortic valve replacement.^1-4^ However, the presence of severe concomitant peripheral artery disease (PAD) can present significant technical challenges for transfemoral access (TFA). In such cases, percutaneous transluminal angioplasty (PTA) may be required to enable TAVI via TFA route, or alternative non-femoral access routes may be considered, including transthoracic access (TTA) or non-femoral, non-thoracic alternative access (nTAA).^5^

The optimal antiplatelet regimen following TAVI remains a topic of considerable debate and clinical investigation.^6,7^ While dual antiplatelet therapy (DAPT) was initially recommended to mitigate thrombotic complications, emerging evidence from randomized controlled trials suggests that single antiplatelet therapy (SAPT) may enhance safety without compromising efficacy in patients undergoing TAVI without concomitant atrial fibrillation (AF).^8^

However, the comparative safety and efficacy of SAPT vs. DAPT in TAVI patients with concomitant severe PAD remain unexplored. Notably, severe PAD is a hallmark of systemic and aggressive atherosclerotic disease, marked by a heightened risk of thrombotic events and adverse clinical outcomes.^9^ This complex condition often necessitates the implementation of more intensive antithrombotic strategies to improve patient prognosis.^10,11^ Moreover, a short course of DAPT is usually recommended in PAD patients undergoing PTA.^7^ However, PAD patients frequently present with multiple comorbidities and a frail overall status, substantially increasing their bleeding risk, particularly when treated with more intensive antithrombotic regimens.^7^ Striking a balance between reducing thrombotic risk and minimizing bleeding complications in patients with severe PAD undergoing TAVI represents a significant clinical challenge.

The HOSTILE registry was a multicentre international study that collected data from 1707 patients with severe PAD undergoing TAVI.^5^ In this study, we sought to provide a dedicated analysis of this large registry focusing on antithrombotic therapy in this high-risk cohort of patients treated through the transfemoral approach or through non-thoracic transalternative approach. Specifically, we compared the safety and efficacy of SAPT vs. DAPT following TAVI in individuals with severe PAD without concomitant AF treated through TFA or nTAA.

## Methods

### Study design and objectives

HOSTILE was a multicentre, international study, retrospectively (from January 2015 to June 2021) and prospectively (from June 2021 to February 2022) collecting data on consecutive patients undergoing TAVI with ‘hostile femoral access’. Hostile femoral access was defined as severe bilateral iliofemoral PAD necessitating pre-procedural PTA (e.g. angioplasty, stenting, or lithotripsy) to enable TAVI via femoral access or requiring alternative (non-femoral) access routes. Patients were categorized into three groups based on access route: TFA (in which pre-procedural PTA was performed to enable TAVI), nTAA (using transaxillary, transcarotid, transbrachiocephalic, or transcaval access), and TTA access (using transapical or transaortic access). Given the limited contemporary use of TTA, this study focused exclusively on patients treated with TFA and nTAA. The main exclusion criteria were unplanned PTA or stenting as a rescue or bailout procedure, aortic aneurysm, large thrombus in the aorta, or TAVI via TFA not requiring PTA despite severe PAD. The Hostile score, ranging from 0 to 39, was used for the assessment of iliofemoral PAD complexity. Patients were classified as having low PAD complexity with a score ≤8.5 and high PAD complexity with a score >8.5 as previously reported.^5^ Details on the inclusion and exclusion criteria as well as on the study design have been previously published.^5^ Data were collected under the rules and governance of the local ethics committee for use of de-identified data. We compared the clinical outcomes of patients undergoing SAPT or DAPT stratified according to main access route and PAD complexity.

### Study endpoints

The primary efficacy endpoint was the propensity-adjusted rate of major adverse cardiovascular events (MACE), a composite of CV death, myocardial infarction (MI), stroke, and transient ischaemic attack (TIA). The primary safety endpoint was the propensity-adjusted rate of major bleeding. Secondary endpoints included any bleeding and all-cause death. All the endpoints were assessed at 30-day and 12-month follow-up and defined according to the Valve Academic Research Consortium 3 definition.^12^

### Statistical analysis

Normal distribution of the data was assessed using the Shapiro–Wilk test. Continuous variables were reported as median and first–third quartile [inter-quartile range (IQR)] and compared using the Mann–Whitney test. Categorical variables were reported as absolute number and frequencies and compared with χ^2^ test or Fisher’s exact test as appropriate. To balance the baseline characteristics between the compared groups, inverse probability of treatment weighting (IPTW) based on the propensity score matching (PSM) was applied. In this method, each individual is assigned a weight derived from their propensity score, which is then used to create a weighted pseudo-population. This process balances confounding variables across comparison groups, aligning baseline characteristics and reducing potential bias. Notably, in the pseudo-population produced through IPTW, summary statistics are based on the total of assigned weights rather than raw patient counts. One notable benefit of IPTW compared with traditional PSM is its ability to retain a larger proportion of the study cohort. Unlike PSM, which often excludes unmatched individuals, IPTW preserves sample size and statistical power, particularly in cases with limited group overlap. The following variables were included in the PS model: age, gender, hypertension, diabetes, prior stroke, prior percutaneous coronary intervention (PCI), prior MI, prior coronary artery bypass grafting (CABG), left ventricular ejection fraction (LVEF), baseline serum creatinine, MI, New York Heart Association (NYHA) class, Hostile score, renal dysfunction, coronary artery disease, body mass index, Society of Thoracic Surgeons score, aortic valve area pre-TAVI, main vascular access used for TAVI, type of valve, residual paravalvular leak, left main coronary artery disease, Euroscore II, use of closure device, haemoglobin levels pre-procedure, and three-vessel coronary artery disease. Observations falling outside the common support were excluded. Only baseline and intraoperative covariates with missing rate <10% were retained for PS matching. Area under curve was calculated for PS models. Absolute standardized mean differences (ASMD) were reported in order to assess balancing across groups. Maximum pairwise ASMD was used to account for the worst unbalanced comparison. Variables with ASMD <0.2 were considered as balanced.^13^ Weighted Kaplan–Meier curves and weighted log-rank test were reported for events at follow-up, and weights were derived from IPTW. Weighted univariate Cox regression models were applied, and Schoenfeld residuals were analysed to verify the proportional hazards assumption. A multivariable logistic regression model considering all baseline and intraoperative characteristics was built to obtain the weights for the inverse IPTW. Hazard ratios (HRs) were reported with 95% confidence intervals (CIs). A two-sided *P*-value of <0.05 was considered statistically significant. Among patients undergoing SAPT, a secondary analysis comparing acetylsalicylic acid (ASA) vs. P2Y_12_ inhibitor monotherapy was performed. As part of the secondary analyses, two subgroups analysis according to the baseline Hostile score (> 8.5 vs. < 8.5) and the main vascular access used to perform TAVI (nTAA vs. TFA) were run. All analyses were performed with STATA 18.0 SE (StataCorp LLC).

## Results

### Antithrombotic therapy after transcatheter aortic valve implantation in patients with severe peripheral artery disease without atrial fibrillation

**Figure 1:**
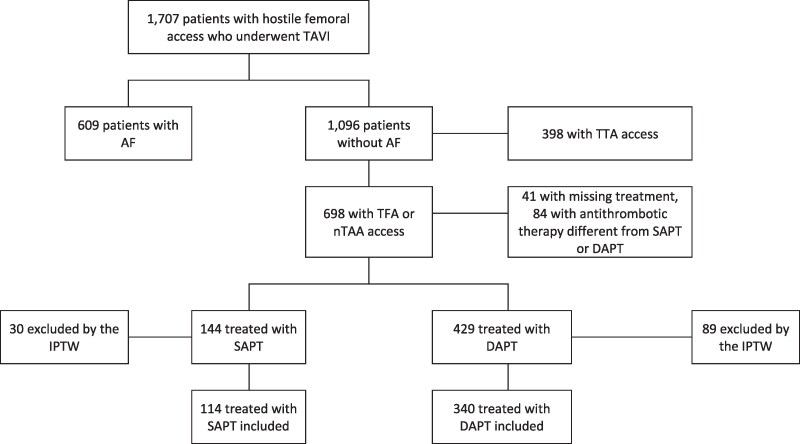
Study flowchart. TAVI, transcatheter aortic valve implantation; AF, atrial fibrillation; DAPT, dual antiplatelet therapy; IPTW, inverse probability of treatment weighting; nTAA, non-thoracic alternative access (non-femoral, non-thoracic); SAPT, single antiplatelet therapy; TFA, transfemoral access; TTA, transthoracic access.

**Figure 2:**
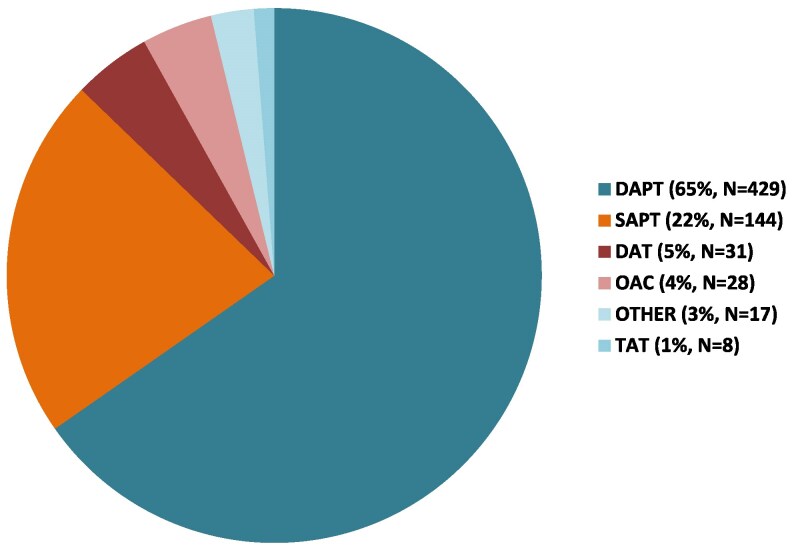
Antithrombotic therapy regimens used after transcatheter aortic valve implantation. DAPT, dual antiplatelet therapy; DAT, dual antithrombotic therapy; OAC, oral anticoagulant therapy; SAPT, single antiplatelet therapy; TAT, triple antithrombotic therapy.

Between January 2015 and February 2022, a total of 1707 consecutive patients with hostile femoral access underwent TAVI in 28 international centres and were included in the study. Of them, 609 had AF, while 1096 did not (*Figure 1*). Among the 1096 patients without AF, 398 patients who underwent TTA were excluded. Of them, 41 (5.9%) were excluded due to missing antithrombotic therapy data and 84 (12%) underwent antithrombotic therapy different from SAPT or DAPT (*Figure 1*). Specifically, 28 (4.3%) were treated with oral anticoagulation (OAC), 31 (4.7%) with OAC plus SAPT [dual antithrombotic therapy (DAT)], 8 (1.2%) with OAC plus DAPT [triple antithrombotic therapy (TAT)], and 17 (2.6%) with other antithrombotic therapy (*Figure 2*). Antithrombotic therapy used in the remaining 573 patients included SAPT in 144 (25.1%) patients—99 of whom underwent ASA and 45 underwent a P2Y_12_ inhibitor—and DAPT in 429 (74.9%) patients, including ASA plus a P2Y_12_ inhibitor. Baseline characteristics were similar between patients excluded due to missing data on antithrombotic therapy and those included in the SAPT/DAPT groups (see Supplementary material online, *Table S1*).

### Clinical outcomes of single vs. dual antiplatelet therapy

Baseline demographic and clinical characteristics of patients receiving SAPT or DAPT are presented in *Table 1*, and procedural characteristics are detailed in *Table 2*. Overall, TFA was used in 273 (47.6%) and nTAA in 300 (52.4%) patients. A moderate-to-severe residual paravalvular leak was observed in 28 (4.9%) patients. Compared with patients undergoing SAPT, those undergoing DAPT were more often affected by coronary artery disease and had undergone prior coronary revascularization more frequently. Dual antiplatelet therapy was more commonly prescribed than SAPT in patients undergoing TAVI via nTAA access routes, while DAPT and SAPT use was similar in those undergoing TAVI via TFA route. Other characteristics, including Hostile score, Euroscore II, NYHA class, left ventricular ejection fraction, pre-procedural haemoglobin levels, and renal function, were similar among the two groups.

**Table 1:** Demographic and clinical characteristics in patients with single or dual antiplatelet therapy

	SAPT	DAPT	Overall	*P*-value	ASMD	ASMD IPTW
***n***	144	429	573			114 VS 340
Age (years), median (IQR)	80.0 (76.0–84.0)	82.0 (76.0–85.0)	81.0 (76.0–85.0)	0.087	0.109	0.09
Male, *n* (%)	68 (47.2)	243 (56.6)	311 (54.3)	0.054	0.189	0.03
Hypertension, *n* (%)	119 (83.2)	385 (90.2)	504 (88.4)	0.034	0.205	0.03
Diabetes, *n* (%)	41 (28.7)	165 (38.5)	206 (36.0)	0.035	0.208	0.11
Three-vessel disease, *n* (%)	13 (9.4)	87 (21.5)	100 (18.5)	0.001	0.339	0.09
Left main coronary artery disease, *n* (%)	6 (5.4)	50 (15.7)	56 (13.0)	0.005	0.340	/
Prior stroke, *n* (%)	12 (8.3)	21 (4.9)	33 (5.8)	0.147	0.138	0.003
Prior PCI, *n* (%)	36 (25.0)	200 (46.6)	236 (41.2)	<0.001	0.462	0.08
Prior MI, *n* (%)	26 (18.1)	128 (29.8)	154 (26.9)	0.006	0.278	0.04
Prior CABG, *n* (%)	17 (11.8)	89 (20.7)	106 (18.5)	0.018	0.243	0.09
Baseline serum creatinine mg/dL, median (IQR)	1.1 (0.8–1.5)	1.1 (0.9–1.4)	1.1 (0.9–1.4)	0.716	0.118	0.01
NYHA class, *n* (%)				0.572	0.060	0.04
I	0 (0.0)	7 (1.6)	7 (1.2)			
II	48 (33.8)	143 (33.5)	191 (33.6)			
III	82 (57.7)	244 (57.1)	326 (57.3)			
IV	12 (8.5)	33 (7.7)	45 (7.9)			
Euroscore II, median (IQR)	5.3 (3.5–8.5)	5.4 (3.5–8.8)	5.3 (3.5–8.8)	0.835	0.082	/
Hostile score, median (IQR)	8.0 (5.5–10.0)	8.0 (6.0–9.5)	8.0 (6.0–9.5)	0.975	0.033	0.12
Renal dysfunction (cr > 1.2 mg/dL), *n* (%)	60 (42.0)	177 (41.3)	237 (41.4)	0.922	0.014	0.06
Coronary artery disease, *n* (%)	60 (41.7)	292 (68.1)	352 (61.4)	<0.001	0.549	0.03
BMI, median (IQR)	25.0 (23.0–28.0)	25.0 (23.0–28.0)	25.0 (23.0–28.0)	0.416	0.117	0.07
STS score, median (IQR)	4.2 (2.7–5.9)	4.8 (3.2–6.9)	4.6 (3.1–6.5)	0.002	0.318	0.06
Aortic valve area pre-TAVI cm^2^, median (IQR)	0.7 (0.6–0.8)	0.7 (0.6–0.8)	0.7 (0.6–0.8)	0.395	0.004	0.002
LVEF, median (IQR)	55.0 (50.0–60.0)	55.0 (45.0–60.0)	55.0 (45.0–60.0)	0.094	0.152	0.07

ASMD, absolute standardized mean differences; BMI, body mass index; CABG, coronary artery bypass grafting; DAPT, dual antiplatelet therapy; IPTW, inverse probability of treatment weighting; LVEF, left ventricular ejection fraction; MI, myocardial infarction; NYHA, New York Heart Association; PCI, percutaneous coronary intervention; SAPT, single antiplatelet therapy; STS score, Society of Thoracic Surgeons Score; TAVI, transcatheter aortic valve implantation.

**Table 2:** Procedural characteristics in patients with single or dual antiplatelet therapy

	SAPT	DAPT	Overall	*P*-value	ASMD	ASMD IPTW
*N*	144	429	573			114 VS 340
Main access, *n* (%)				0.50	0.07	0.03
Femoral	65 (45.1)	208 (48.5)	273 (47.6)			
Alternative	79 (54.9)	221 (51.5)	300 (52.4)			
Echo guided puncture, *n* (%)	40 (29.9)	125 (31.6)	165 (31.2)	0.75	0.04	/
Type of valve, *n* (%)				<0.001	0.14	0.04
Accurate	6 (4.2)	6 (1.4)	12 (2.1)			
Evolut/Corevalve	64 (44.4)	255 (59.4)	319 (55.7)			
Portico/Navitor	34 (23.6)	16 (3.7)	50 (8.7)			
Sapien	29 (20.1)	141 (32.9)	170 (29.7)			
Symetis	8 (5.6)	9 (2.1)	17 (3.0)			
Other	2 (1.4)	1 (0.2)	3 (0.5)			
Mean aortic valve gradient post-TAVI (mmHg), median (IQR)	8.0 (6.0–11.0)	8.0 (5.0–11.0)	8.0 (6.0–11.0)	0.64	0.05	0.06
Residual paravalvular leak, *n* (%)				0.36	0.04	<0.001
None	76 (52.8)	244 (57.0)	320 (55.9)			
Trivial/mild	63 (43.8)	161 (37.6)	224 (39.2)			
Moderate/severe	5 (3.5)	23 (5.4)	28 (4.9)			
Closure device, *n* (%)				0.02	0.10	/
1 ProGlide	10 (6.9)	13 (3.0)	23 (4.0)			
2 ProGlides	44 (30.6)	147 (34.3)	191 (33.3)			
> 2 ProGlides	0 (0.0)	12 (2.8)	12 (2.1)			
Angioseal	0 (0.0)	11 (2.6)	11 (1.9)			
Covered stent	0 (0.0)	2 (0.5)	2 (0.3)			
Combination of device	11 (7.6)	36 (8.4)	47 (8.2)			
Manta	15 (10.4)	21 (4.9)	36 (6.3)			
Prostar	7 (4.9)	11 (2.6)	18 (3.1)			
Surgical	41 (28.5)	116 (27.0)	157 (27.4)			
No	0 (0.0)	0 (0.0)	0 (0.0)			
Haemoglobin levels pre-procedure (g/dL), median (IQR)	11.5 (10.0–12.9)	12.0 (10.5–13.0)	11.9 (10.4–13.0)	0.21	0.15	/

ASMD, absolute standardized mean differences; DAPT, dual antiplatelet therapy; IPTW, inverse probability of treatment weighting; SAPT, single antiplatelet therapy; TAVI, transcatheter aortic valve implantation.

Area under the receiver operating characteristic curve was equal to 0.76 (95% CI 0.71–0.81), resulting in a good model discrimination. Patients with complete data and falling within the common support area were 79.2% of the overall initial cohort (454/573), with similar percentages among SAPT and DAPT groups (79.2% and 79.3%, respectively).

**Figure 3:**
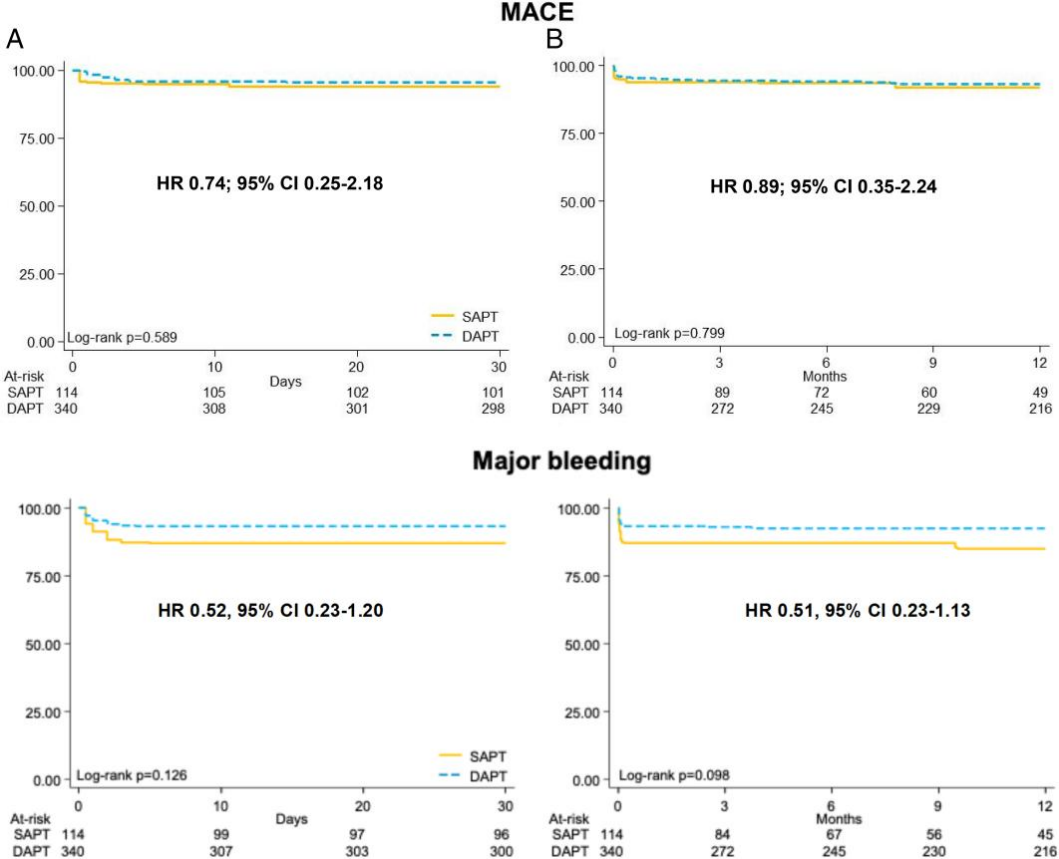
Weighted Kaplan–Meier curves of major adverse cardiovascular events and major bleeding at 30 days (*A*) and 12 months (*B*). DAPT, dual antiplatelet therapy; MACE, major adverse cardiovascular events; HR, hazard ratio; CI, confidence interval; SAPT, single antiplatelet therapy.

**Figure 4:**
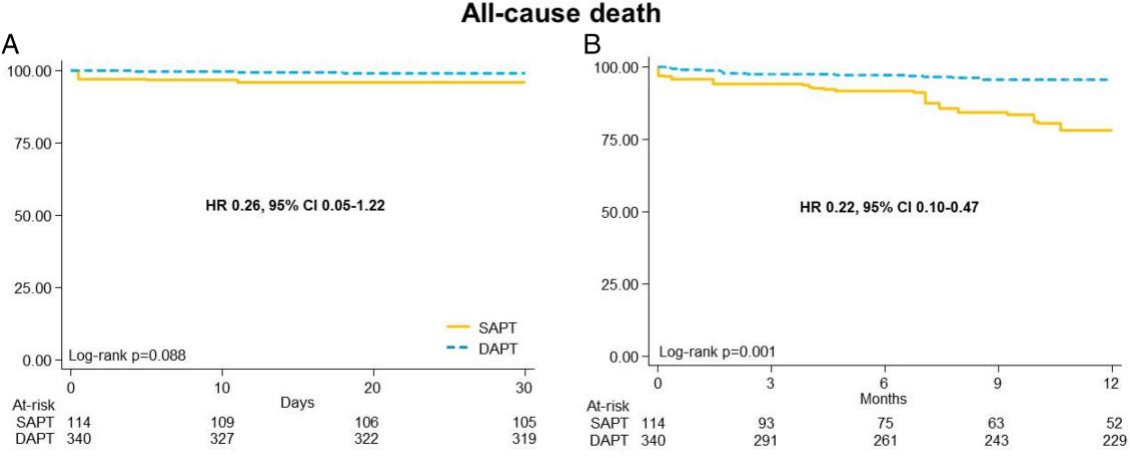
Weighted Kaplan–Meier curves of all-cause death at 30 days (*A*) and 12 months (*B*). DAPT, dual antiplatelet therapy; MACE, major adverse cardiovascular events; HR, hazard ratio; CI, confidence interval; SAPT, single antiplatelet therapy.

Weighted Kaplan–Meier curves are reported in *Figures 3* and *4* and Supplementary material online, *Figure S1*. There was a non-significant reduction in MACE at 30 days (HR 0.74, 95% CI: 0.25–2.18; *P* = 0.59) and 12 months (HR 0.89, 95% CI 0.35–2.24; *P* = 0.80) with DAPT compared with SAPT (*Table 3*). There was no difference in major bleeding at 30 days (*P* = 0.13) or 12 months (*P* = 0.10) between groups (*Figure 3* and *Table 3*). There was a significant reduction in all-cause death with DAPT compared with SAPT at both 12 months (HR 0.22, 95% CI: 0.10–0.47; *P* < 0.001) but not at 30 days (HR 0.26, 95% CI: 0.05–1.22; *P* = 0.09) (*Figure 3* and *Table 3*). No significant difference was found between groups in any bleeding at 30 days (*P* = 0.16) or 12 months (*P* = 0.17) (*Table 3* and Supplementary material online, *Figure S1*). Individual and composite events at 30 days and at 12 months are reported in Supplementary material online, *Table S2*.

**Table 3:** Weighted univariate Cox regression models of DAPT vs. SAPT

	HR	95% CI		*P*-value
MACE (CV death, MI, stroke, or TIA)				
30 days	0.74	0.25	2.18	0.59
12 months	0.89	0.35	2.24	0.80
Major bleeding				
30 days	0.52	0.23	1.20	0.13
12 months	0.51	0.23	1.13	0.10
Any bleeding				
30 days	0.61	0.31	1.23	0.16
12 months	0.63	0.33	1.21	0.17
All-cause death				
30 days	0.26	0.05	1.22	0.09
12 months	0.22	0.10	0.47	<0.001

DAPT, dual antiplatelet therapy; MACE, major adverse cardiovascular events; MI, myocardial infarction; SAPT, single antiplatelet therapy; TIA, transient ischaemic attack; HR, hazard ratio; CI, confidence interval; CV, cardiovascular.

In the secondary analyses, the use of ASA vs. P2Y_12_ inhibitor monotherapy in the SAPT group showed no significant differences between the groups for any of the evaluated outcomes (see Supplementary material online, *Table S3*). Of note, a significant interaction was observed between antiplatelet therapy and the Hostile score for the outcome of MACE, with DAPT associated with better clinical outcomes in patients with Hostile score > 8.5 compared with those with a score ≤ 8.5 (*P*_interaction_ = 0.01 at both 30 days and 12 months). (see Supplementary material online, *Table S4*). Finally, there were no differences in any of the included outcomes between DAPT vs. SAPT in the subgroups of patients undergoing TAVI via TFA or nTAA (see Supplementary material online, *Table S4*).

## Discussion

This study is the largest to date examining the comparative outcomes of SAPT vs. DAPT in TAVI patients with severe PAD. The key findings of this analysis can be summarized as follows: (i) significant heterogeneity exists in the choice of antithrombotic therapy for TAVI patients with severe PAD, with 18% of individuals receiving antithrombotic regimens that deviate from the recommended SAPT or DAPT protocols; (ii) compared with SAPT, DAPT was associated with a non-significant reduction in MACE at both 30 days and 12 months, with a significant interaction between antiplatelet therapy and the Hostile score, suggesting a greater benefit of DAPT in patients with high PAD complexity; (iii) DAPT was associated with reduced all-cause mortality at 12 months compared with SAPT; and (iv) in the SAPT group, the choice between ASA or a P2Y_12_ inhibitor did not result in significant differences in outcomes.

Dual antiplatelet therapy with ASA and a P2Y_12_ inhibitor represents the standard of care for patients undergoing PCI^14^ and was initially adopted empirically for other cardiac and non-cardiac percutaneous interventions, including TAVI.^7,15^ However, DAPT is associated with an elevated risk of bleeding compared with SAPT, which carries significant clinical implications and, in certain scenarios, may outweigh its benefits in reducing thrombotic events.^16,17^ Additionally, the effectiveness of antithrombotic therapy is significantly impacted by clinical, procedural, demographic, and genetic factors, highlighting the importance of tailoring treatment to each patient to achieve an optimal balance between bleeding and ischaemic risks.^18^

Recent evidence suggests that, for TAVI patients without an indication for OAC, such as those without AF, SAPT may offer an optimal balance between thrombotic and bleeding risks.^8^ In fact, SAPT has been shown to reduce thrombotic events to a similar extent as DAPT, while minimizing the risk of bleeding.^8^ However, no evidence on the comparative effects of SAPT vs. DAPT is available in the specific setting of patients with severe PAD undergoing TAVI.

Severe PAD is a clinical condition marked by a high thrombotic burden and an elevated risk of CV events, as PAD serves as a marker of diffuse and aggressive systemic atherosclerotic disease.^9^ Therefore, more intense antithrombotic therapy has been shown to reduce the risk of limb amputation and revascularization as well as of CV events at the cost of increased risk of bleeding.^19^ However, patients with PAD also tend to be characterized by frailty and a heightened bleeding risk, further complicating the optimization of antithrombotic therapy in this vulnerable population.^20^

The management of antithrombotic therapy in patients with severe PAD undergoing TAVI presents a particularly challenging clinical scenario. This complexity arises from the dual challenges surrounding the optimal antithrombotic regimen after TAVI and the appropriate strategy for PAD patients, whether they undergo PTA or not.

In this study, we observed that nearly one in five patients with severe PAD undergoing TAVI received an antithrombotic therapy that deviated from the standard recommendations for patients without concomitant AF, traditionally consisting of SAPT or DAPT.^21^ This emphasizes the absence of specific recommendations and the substantial clinical challenges in defining the optimal antithrombotic regimen for this high-risk patient population.

When comparing SAPT vs. DAPT at 30 days and 12 months, we found that DAPT was associated with no difference in the primary efficacy endpoints. However, there was a significant interaction between antiplatelet therapy and the Hostile score, suggesting that the benefit of DAPT may be more pronounced in patients with more severe PAD. These findings should be interpreted considering the study’s relatively small sample size and the potential limitations in statistical power for assessing the included endpoints. Of note, we observed a significant reduction in 1-year all-cause mortality with DAPT vs. SAPT, a finding of uncertain interpretation that warrants additional investigation.

Interestingly, we observed no significant differences in event rates between ASA and P2Y_12_ inhibitors in patients treated with SAPT. Although these findings are limited by the limited sample size, they suggest that ASA and P2Y_12_ inhibitors may offer comparable effectiveness in this high-risk population. Furthermore, in our analysis, the access site utilized for TAVI in patients with severe PAD does not seem to influence the safety and efficacy of the subsequent antiplatelet therapy. These findings are exploratory and require further validation through additional evidence.

Although subject to several limitations, this study represents the largest to date focusing on the relatively rare and high-risk subgroup of patients with severe PAD undergoing TAVI. Acknowledging that no specific recommendation can be provided by a non-randomized study, our findings call for further investigations in view of the potential benefits that DAPT may offer especially in patients with more severe PAD.

### Limitations

This is a retrospective and prospective observational study and, as such, carries all the limitations inherent to non-randomized research. Consequently, these findings should be interpreted as hypothesis generating. Moreover, the statistical power may be insufficient to adequately assess outcomes during follow-up. However, this remains the largest study conducted to date in this relatively rare yet high-risk clinical setting. We were unable to reliably assess adherence to antiplatelet therapy or the exact duration of DAPT beyond 30 days. As such, our study does not allow for conclusions regarding the optimal length of DAPT in TAVI patients with severe PAD. Finally, detailed information on the causes of non-cardiac deaths, as well as PAD-related outcomes such as acute limb ischaemia or major amputations due to vascular causes, was not captured in this study.

## Conclusions

This study suggests that an initial course of DAPT following TAVI may be of potential benefit in patients with more severe PAD in terms of MACE and mortality reduction. Further evidence is needed to validate these findings and establish the optimal duration of DAPT after TAVI in this high-risk population.

## Supplementary Material

pvaf049_Supplementary_Data

## Data Availability

The data underlying this article will be shared on reasonable request to the corresponding author.
