# Supplementary material for: Single vs. dual antiplatelet therapy in patients with severe peripheral arterial disease undergoing transcatheter aortic valve implantation: insights from the Hostile registry
Source: Eur Heart J Cardiovasc Pharmacother. 2025 Jul 15;11(6):524–31. doi: 10.1093/ehjcvp/pvaf049 (PMC12450595; doi:10.1093/ehjcvp/pvaf049)
Supplement: pvaf049_Supplementary_Data [file pvaf049_supplementary_data.docx]

**SUPPLEMENTARY APPENDIX**

**Single versus Dual Antiplatelet Therapy in Patients with Severe Peripheral Arterial Disease Undergoing TAVI: Insights from The Hostile Registry**

Galli et al.

**Table of contents**

**Supplementary Figure 1:** Weighted Kaplan Meier curves of any bleeding at 30 days (A) and 12 months (B).

**Supplementary Table 1:** Baseline characteristics of patients excluded because of missing data on antithrombotic therapy and patients included in the study (SAPT/DAPT).

**Supplementary Table 2:** Number of events at 30 days and 12 months.

**Supplementary Table 3:** Secondary analysis comparing acetylsalicylic acid versus P2Y_12_ inhibitor monotherapy among patients undergoing single antiplatelet therapy.

**Supplementary Table 4:** Weighted Cox regression models using the Hostile score and main vascular access used to perform TAVI as covariates.

**Supplementary Figure 1:** Weighted Kaplan Meier curves of any bleeding at 30 days (A) and 12 months (B).

**
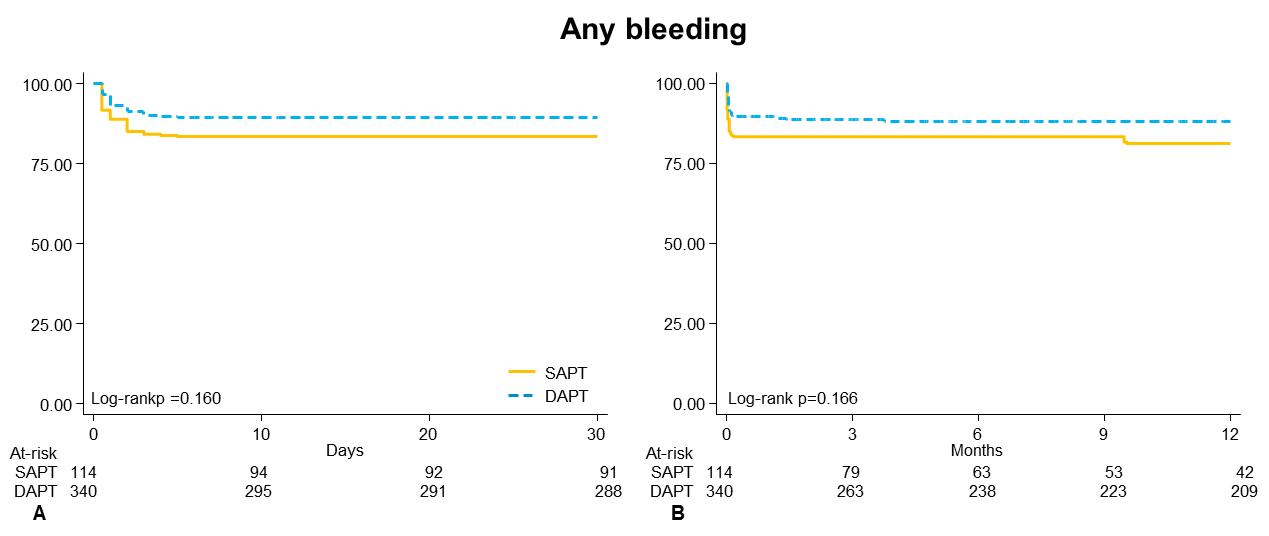
**

Abbreviations: SAPT: single antiplatelet therapy; DAPT: dual antiplatelet therapy; MACE: major adverse cardiovascular events.

**Supplementary Table 1: Baseline characteristics of patients excluded because of missing data on antithrombotic therapy and patients included in the study (SAPT/DAPT).**

|  | Missing | DAPT/SAPT | p |
| --- | --- | --- | --- |
| n | 41 | 573 |  |
| Age (Years), Median (IQR) | 80.2 (74.0, 85.0) | 81.0 (76.0, 85.0) | 0.685 |
| Male, n (%) | 21 (51.2) | 311 (54.3) | 0.747 |
| Hypertension, n (%) | 39 (95.1) | 504 (88.4) | 0.300 |
| Diabetes, n (%) | 9 (22.0) | 206 (36.0) | 0.089 |
| Three Vessel Disease, n (%) | 4 (15.4) | 100 (18.5) | 1.000 |
| Left Main Coronary Artery Disease, n (%) | 1 (5.0) | 56 (13.0) | 0.492 |
| Prior Stroke, n (%) | 2 (4.9) | 33 (5.8) | 1.000 |
| Prior PCI, n (%) | 17 (41.5) | 236 (41.2) | 1.000 |
| Prior MI, n (%) | 11 (26.8) | 154 (26.9) | 1.000 |
| Prior CABG, n (%) | 5 (12.2) | 106 (18.5) | 0.403 |
| Baseline Serum Creatinine (Mg/Dl), Median (IQR) | 1.11 (0.79, 1.60) | 1.11 (0.86, 1.40) | 0.789 |
| NYHA Class, n (%) |  |  | <0.001 |
| 1 | 5 (12.2) | 7 (1.2) |  |
| 2 | 10 (24.4) | 191 (33.6) |  |
| 3 | 18 (43.9) | 326 (57.3) |  |
| 4 | 8 (19.5) | 45 (7.9) |  |
| Euroscore II, Median (IQR) | 4.66 (2.77, 9.40) | 5.34 (3.50, 8.82) | 0.268 |
| Hostile Score, Median (IQR) | 9.0 (8.0, 10.0) | 8.0 (6.0, 9.5) | 0.013 |
| Hostile Score>8.5, n (%) | 25 (61.0) | 238 (41.6) | 0.021 |
| Renal Dysfunction (Cr>1.2mg/Dl), n (%) | 17 (41.5) | 237 (41.4) | 1.000 |
| Coronary Artery Disease, n (%) | 27 (65.9) | 352 (61.4) | 0.621 |
| BMI, Median (IQR) | 25.0 (24.0, 29.0) | 25.0 (23.0, 28.0) | 0.742 |
| STS Score, Median (IQR) | 4.46 (3.26, 6.98) | 4.64 (3.12, 6.49) | 0.766 |
| Aortic Valve Area Pre TAVI (Cm2), Median (IQR) | 0.70 (0.50, 0.80) | 0.70 (0.60, 0.80) | 0.663 |
| Left Ventricular Ejection Fraction, Median (IQR) | 58.0 (39.0, 6500) | 55.0 (45.0, 60.0) | 0.444 |
| Main Access Femoral, n (%) | 14 (34.1) | 273 (47.6) | 0.106 |

Abbreviations: IQR: interquartile range; TAVI: transcatheter aortic valve implantation; NYHA New York heart association; SAPT: single antiplatelet therapy; DAPT: dual antiplatelet therapy; CV: cardiovascular; MACE: major adverse cardiovascular events; MI: myocardial infarction; TIA: transient ischemic attack.

**Supplementary Table 2: Number of events at 30 days and 12 months.**

|  | **SAPT** | **DAPT** | **Overall** |
| --- | --- | --- | --- |
|  | 144 | 429 | 573 |
| **30 DAYS** |  |  |  |
| All-cause Death, n (%) | 4 (2.8) | 11 (2.6) | 15 (2.6) |
| CV Death, n (%) | 4 (2.8) | 7 (1.6) | 11 (1.9) |
| MI, n (%) | 1 (0.7) | 3 (0.7) | 4 (0.7) |
| Stroke, n (%) | 7 (4.9) | 7 (1.6) | 14 (2.4) |
| Stroke/TIA, n (%) | 7 (4.9) | 14 (3.3) | 21 (3.7) |
| Any Bleeding, n (%) | 23 (16.0) | 54 (12.6) | 77 (13.4) |
| Major Bleeding, n (%) | 17 (11.8) | 36 (8.4) | 53 (9.2) |
| MACE (any death, stroke, MI, TIA) | 10 (6.9) | 25 (5.8) | 35 (6.1) |
| MACE (CV death, stroke, MI, TIA) | 10 (6.9) | 21 (4.9) | 31 (5.4) |
| **12 MONTHS** |  |  |  |
| All-cause Death, n (%) | 20 (13.9) | 26 (6.1) | 46 (8.0) |
| CV Death, n (%) | 8 (5.6) | 16 (3.8) | 24 (4.2) |
| MI, n (%) | 1 (0.7) | 7 (1.6) | 8 (1.4) |
| Stroke, n (%) | 7 (4.9) | 8 (1.9) | 15 (2.6) |
| Stroke/TIA, n (%) | 7 (4.9) | 15 (3.5) | 22 (3.8) |
| Any Bleeding, n (%) | 25 (17.4) | 59 (13.8) | 84 (14.7) |
| Major Bleeding, n (%) | 19 (13.2) | 38 (8.9) | 57 (9.9) |
| MACE (any death, stroke, MI, TIA) | 26 (18.1) | 43 (10.0) | 69 (12.0) |
| MACE (CV death, stroke, MI, TIA) | 14 (9.7) | 34 (7.9) | 48 (8.4) |

Abbreviations: SAPT: single antiplatelet therapy; DAPT: dual antiplatelet therapy; CV: cardiovascular; MACE: major adverse cardiovascular events; MI: myocardial infarction; TIA: transient ischemic attack.

**Supplementary Table 3:** Secondary analysis comparing acetylsalicylic acid versus P2Y_12_ inhibitor monotherapy among patients undergoing single antiplatelet therapy.

|  |  | **HR** | **95% CI** | | **P value** |
| --- | --- | --- | --- | --- | --- |
| **MACE** (CV death, MI, stroke or TIA) | | | | | |
| 30 days | DAPT vs SAPT | 0.74 | 0.25 | 2.18 | 0.59 |
|  | Clop vs ASA | 0.64 | 0.06 | 7.04 | 0.71 |
| 12 months | DAPT vs SAPT | 0.89 | 0.35 | 2.24 | 0.80 |
|  | Clop vs ASA | 2.40 | 0.44 | 13.13 | 0.31 |
| **Major Bleeding** | | | | | |
| 30 days | DAPT vs SAPT | 0.52 | 0.23 | 1.20 | 0.13 |
|  | Clop vs ASA | 1.76 | 0.39 | 7.87 | 0.46 |
| 12 months | DAPT vs SAPT | 0.51 | 0.23 | 1.13 | 0.10 |
|  | Clop vs ASA | 1.76 | 0.39 | 7.87 | 0.46 |
| **Any Bleeding** | | | | | |
| 30 days | DAPT vs SAPT | 0.61 | 0.31 | 1.22 | 0.16 |
|  | Clop vs ASA | 1.32 | 0.42 | 4.08 | 0.64 |
| 12 months | DAPT vs SAPT | 0.63 | 0.33 | 1.21 | 0.17 |
|  | Clop vs ASA | 1.32 | 0.42 | 4.08 | 0.64 |
| **All-cause Death** | | | | | |
| 30 days | DAPT vs SAPT | 0.26 | 0.05 | 1.22 | 0.09 |
|  | Clop vs ASA | 1.30 | 0.08 | 20.76 | 0.85 |
| 12 months | DAPT vs SAPT | 0.22 | 0.10 | 0.47 | <0.001 |
|  | Clop vs ASA | 1.01 | 0.34 | 3.01 | 0.99 |

Abbreviations: DAPT: dual antiplatelet therapy, SAPT: single antiplatelet therapy; Clop: clopidogrel; ASA: acetylsalicylic acid; CV: cardiovascular; MI: Myocardial infarction; MACE: major adverse cardiovascular events; HR: hazard ratio; CI: confidence interval.

**Supplementary Table 4:** Weighted Cox regression models using the Hostile score and main vascular access used to perform TAVI as covariates.

| **MACE (CV death, MI, stroke, TIA)** | | | | | |
| --- | --- | --- | --- | --- | --- |
| 30 days | HR | 95% CI | | p | p for interaction |
| DAPT vs SAPT | 0.74 | 0.25 | 2.18 | 0.59 |  |
| Hostile ≤8.5 | 1.80 | 0.33 | 9.88 | 0.50 | 0.01 |
| Hostile >8.5 | 0.54 | 0.15 | 1.90 | 0.34 |  |
| nTAA | 0.53 | 0.15 | 1.85 | 0.32 | 0.36 |
| TFA | 1.77 | 0.21 | 14.86 | 0.60 |  |
| 12 months | HR | 95% CI | | P | p for interaction |
| DAPT vs SAPT | 0.89 | 0.35 | 2.24 | 0.80 |  |
| Hostile ≤8.5 | 1.03 | 0.27 | 3.89 | 0.97 | 0.02 |
| Hostile >8.5 | 0.86 | 0.26 | 2.82 | 0.81 |  |
| nTAA | 0.73 | 0.22 | 2.40 | 0.60 | 0.74 |
| TFA | 1.16 | 0.28 | 4.79 | 0.83 |  |
| **Major bleeding** | | | | | |
| 30 days | HR | 95% CI | | P | p for interaction |
| DAPT vs SAPT | 0.52 | 0.23 | 1.20 | 0.13 |  |
| Hostile ≤8.5 | 0.74 | 0.120 | 2.74 | 0.65 | 0.86 |
| Hostile >8.5 | 0.35 | 0.11 | 1.05 | 0.06 |  |
| nTAA | 0.30 | 0.11 | 0.84 | 0.02 | 0.25 |
| TFA | 1.16 | 0.26 | 5.23 | 0.85 |  |
| 12 months | HR | 95% CI | | P | p for interaction |
| DAPT vs SAPT | 0.51 | 0.23 | 1.13 | 0.10 |  |
| Hostile ≤8.5 | 0.63 | 0.19 | 2.08 | 0.45 | 0.97 |
| Hostile >8.5 | 0.41 | 0.14 | 1.18 | 0.10 |  |
| nTAA | 0.29 | 0.10 | 0.80 | 0.02 | 0.33 |
| TFA | 1.04 | 0.28 | 3.82 | 0.96 |  |
| **Any bleeding** | | | | | |
| 30 days | HR | 95% CI | | P | p for interaction |
| DAPT vs SAPT | 0.61 | 0.31 | 1.22 | 0.16 |  |
| Hostile ≤8.5 | 0.69 | 0.25 | 1.87 | 0.46 | 0.96 |
| Hostile >8.5 | 0.54 | 0.21 | 1.40 | 0.21 |  |
| nTAA | 0.41 | 0.17 | 0.98 | 0.05 | 0.29 |
| TFA | 1.05 | 0.34 | 3.23 | 0.93 |  |
| 12 months | HR | 95% CI | | P | p for interaction |
| DAPT vs SAPT | 0.63 | 0.33 | 1.21 | 0.17 |  |
| Hostile ≤8.5 | 0.67 | 0.26 | 1.71 | 0.40 | 0.95 |
| Hostile >8.5 | 0.59 | 0.24 | 1.48 | 0.26 |  |
| nTAA | 0.39 | 0.17 | 0.94 | 0.04 | 0.48 |
| TFA | 1.07 | 0.39 | 2.96 | 0.90 |  |
| **All-cause death** | | | | | |
| 30 days | HR | 95% CI | | P | p for interaction |
| DAPT vs SAPT | 0.26 | 0.05 | 1.22 | 0.09 |  |
| Hostile ≤8.5 | 1.18 | 0.12 | 11.58 | 0.88 | 0.46 |
| Hostile >8.5 | 0.08 | 0.01 | 0.82 | 0.03 |  |
| nTAA | 0.14 | 0.02 | 0.88 | 0.04 | 0.13 |
| TFA | / | / | / | / |  |
| 12 months | HR | 95% CI | | p | p for interaction |
| DAPT vs SAPT | 0.22 | 0.10 | 0.47 | <0.001 |  |
| Hostile ≤8.5 | 0.16 | 0.05 | 0.52 | 0.002 | 0.86 |
| Hostile >8.5 | 0.28 | 0.10 | 0.82 | 0.02 |  |
| nTAA | 0.31 | 0.11 | 0.86 | 0.02 | 0.03 |
| TFA | 0.13 | 0.04 | 0.43 | 0.001 |  |

Abbreviations: ASA: acetylsalicylic acid; DAPT: dual antiplatelet therapy; MACE: major adverse cardiovascular events; MI: myocardial infarction; NACE: net adverse clinical events; SAPT: single antiplatelet therapy; nTAA: non-thoracic transalternative access; TFA: transfemoral access; TIA: transient ischemic attack; TTA: transthoracic access.
